# Brain imaging research is thriving in functional neurological disorder

**DOI:** 10.1016/j.nicl.2026.104004

**Published:** 2026-05-16

**Authors:** David L. Perez, Kasia Kozlowska, Selma Aybek

**Affiliations:** a Mass General Brigham Departments of Neurology and Psychiatry, Massachusetts General Hospital and Brigham and Women’s Hospital, Harvard Medical School, Boston, MA, USA; b The Children’s Hospital at Westmead Clinical School; Discipline of Psychiatry and Discipline of Child & Adolescent Health, University of Sydney Medical School, Sydney, Australia; c Westmead Institute for Medical Research, Westmead, NSW, Australia; d Neurology, Science and Medicine Faculty, Fribourg University, Fribourg, Switzerland

## 1 Introduction

Functional neurological disorder (FND) is a condition at the neurology-psychiatry interface that impacts individuals across the lifespan (Hallett et al., 2022). The conceptualization of FND in the 20th century was fraught with dualistic perspectives of brain versus mind – exemplified by outdated terms such as “hysteria” and “psychogenic”. Structure and function are closely coupled in the human brain, and neuroimaging research in FND provides an opportunity to bridge the science of brain and mind in this prevalent and potentially disabling condition (Finkelstein et al., 2025, O’Mahony et al., 2023). Advancing the neuroscience of FND further erodes artificial distinctions between brain health and mental health – and offers the potential to develop novel mechanistically-informed treatments.

The first neuroimaging studies in FND were published in the late 1990s – and the reconceptualization of FND as a rule in diagnosis, using clinical bedside signs specific for the condition, has catalyzed renewed scientific interest (Aybek and Perez, 2022, Perez et al., 2021). While the field of FND needs better support from healthcare systems, funding agencies, and policy makers, pathophysiology research in FND is thriving. As such, *NeuroImage: Clinical* commissioned a Special Issue on this topic. Consistent with a multimodal approach to the neurobiological study of FND, this twelve-article collection includes five functional magnetic resonance imaging (fMRI) studies (Bleier et al., 2026, Müller et al., 2025, Premi et al., 2025, Voženílek et al., 2026, Walpola et al., 2025), five structural MRI (sMRI) studies (Gninenko et al., 2025, Helmstaedter et al., 2025, Lan et al., 2025, Sharma et al., 2025, Westlin et al., 2025), one resting-state electroencephalography study (Lozzi et al., 2026), and an article on the utility of ultra-high field strength MRI to delineate the neurocircuitry of FND (Myren et al., 2026).

## 2 fMRI studies

Voženílek et al. (2026) characterized relationships between static resting-state functional connectivity (rsFC) and pre-pulse inhibition (PPI) of the startle reflex in 38 patients with the motor variant of FND (FND-motor) compared to 39 healthy controls (HCs). PPI size, a measure of sensorimotor gaiting, was reduced in FND-motor vs. HCs. In HCs, seed-based analyses showed that PPI size correlated with insula-to-temporoparietal junction (TPJ) rsFC, as well as with right TPJ-left TPJ connectivity. These PPI size–rsFC relationships were not found in the FND-motor cohort. The authors suggested that findings indicated a potential failure of top-down filtering of sensory information in FND-motor.

Premi et al. (2025) studied rsFC differences in 15 patients with functional hemiparesis (FND-par) versus 52 HCs, including testing relationships between rsFC and GABAergic transmission via short interval intracortical inhibition (SICI) values (obtained from transcranial magnetic stimulation (TMS) applied to the primary motor cortex). There were no findings with static rsFC. In dynamic rsFC analyses compared to HCs, patients with FND-par showed decreased dwell time in state 2 of the somatomotor network, as well as increased dwell time in state 1 of the default mode network and state 3 of the salience network. Among observed findings in the FND-par cohort, shorter state 2 dwell time in the somatomotor network correlated with lower SICI [higher GABAergic transmission]. The authors suggested that altered dynamic rsFC and GABAergic transmission may have mechanistic relevance in FND-par.

Bleier et al. (2026) investigated relationships between static rsFC and resting cardiac autonomic metrics (heart rate (HR), HR variability (HRV)) in 20 females with mixed FND (FND-mixed) and 23 female psychiatric controls (PCs). Compared to PCs, females with FND-mixed showed no HR or HRV differences. In FND-mixed, higher HR correlated with higher weighted-degree centrality in the bilateral supplementary motor areas (SMA); lower HRV correlated with higher weighted-degree centrality in the bilateral SMA, mid-cingulate cortices, temporal poles, and the right anterior insula, lateral orbitofrontal cortex, and amygdala. In PCs, correlations with HRV scores were more spatially restricted. The authors pointed out that functional connectivity – cardiac autonomic associations, implicating regions of the central autonomic, salience, and allostatic-interoceptive networks, help contextualize brain organization heterogeneity commonly found in FND.

Walpola et al. (2025) applied static rsFC in 28 patients with pediatric FND versus 31 HCs to study connectivity differences in interoceptive processing (anchored in the insula), exteroceptive processing (anchored in the TPJ), and mental self-processing (anchored in the anterior medial prefrontal cortex (amPFC)) networks. In the mental self-processing network, children/adolescents with FND showed increased amPFC rsFC to the thalamus and left dorsolateral prefrontal cortex (dlPFC); the other networks did not show group-level differences. Those with functional seizures vs. other FND presentations in the mental-self processing network exhibited decreased amPFC-right TPJ rsFC. In the interoceptive processing network, adverse childhood experience (ACE) burden correlated with left insula coupling to the right TPJ and dorsal anterior cingulate cortex. Resting HR in the pediatric FND cohort showed a trend-level correlation with left intra-insula rsFC strength. The authors highlighted that more research is needed regarding factors that impact the organization of self-processing networks in pediatric FND.

Müller et al. (2025) used real-time fMRI neurofeedback in 18 patients with FND-mixed to attempt to modulate right TPJ activity in the context of receiving intermittent feedback about their right TPJ activity while performing an agency task (i.e., moving a cursor along a horizontal bar to catch targets while avoiding distractors, with some trials having an element of noise [turbulence]). In the first session, patients with FND-mixed that successfully upregulated right TPJ and SMA activity also reported increased self-agency after training. Non-responders to the training showed a decrease in right TPJ-SMA connectivity after training, while responders showed trend-level right TPJ-dlPFC connectivity decreases. The authors established the feasibility of using real-time fMRI to enable some FND patients to adaptively modulate their right TPJ activity – setting the stage for larger-scale, controlled, and longitudinal real-time fMRI studies in FND.

## 3 sMRI studies

Helmstaedter et al. (2025) performed *FreeSurfer* analyses on clinical grade MRI scans in 88 patients with functional seizures compared to 79 neurologically healthy controls. With an *a priori* focus on the precuneus [a posteromedial midline structure implicated in dissociation], the authors identified no between-group differences. However, longer illness duration correlated with relative reductions in left precuneus cortical volume in the functional seizure cohort; a later age of illness onset was also associated with relative reductions in bilateral precuneus volumes. Greater “reduced perception of the self and reality” was associated with right precuneus cortical thickness reductions. The authors underscored the emerging relevance of structural precuneus alterations as associated with functional seizure illness features – including dissociation severity.

Sharma et al. (2025) evaluated the post versus pre-treatment impact of neurobehavioral therapy (NBT) on grey matter in 50 patients with functional seizures *and* a history of traumatic brain injury (TBI); 50 individuals with a TBI and 33 HCs were used as comparison groups. In voxel-based morphometry analyses compared to both control cohorts, individuals with functional seizures showed an increase in left inferior and middle temporal gyrus volume, accompanied by a 35.8 percent reduction in seizure frequency and improvements in depression and anxiety scores. In the functional seizure plus TBI cohort, percent change in inferior temporal gyrus volume correlated with percent change in Global Assessment of Functioning scores. The authors highlighted associations between a positive NBT response and neuroplastic changes in temporal regions.

Westlin et al. (2025) used *FreeSurfer* to study cortical thickness and subcortical volumes in 37 patients with functional cognitive disorder (FND-cognitive) after concussion compared to 25 post-concussion controls. While no between-group sMRI differences were observed, functional memory symptom severity correlated transdiagnostically (n = 62) with relative increases in right amygdala volume – a result driven by differences in lateral, basal, and paralaminar amygdala nuclei. Twenty-four individuals with FND-cognitive also participated in a cognitive behavioral therapy vs. cognitive remediation trial. Adjusting for treatment type and baseline symptom severity, relative pre-treatment increases in right inferior frontal gyrus cortical thickness predicted greater post-treatment improvement. The authors noted that amygdala findings fit well with formulations linking functional cognitive symptoms to biased attentional and heightened threat processing; prefrontal cortical areas are emerging as a transdiagnostic marker of clinical outcomes.

Gninenko et al. (2025) compared diffuse-weighted imaging in 85 patients with FND-mixed and 75 HCs. Weighted-degree fractional anisotropy values for the grey matter origins of white matter fibers were calculated using graph theory and probabilistic tractography. Adjusting for age and sex compared to HCs, the FND-mixed cohort showed reduced microstructural integrity from 19 regions – including the right lateral orbitofrontal cortex, insula, superior temporal gyrus and putamen. These differences were no longer significant [although trends remained] when adjusting for depression and anxiety scores or antidepressant and benzodiazepine use. In the FND-mixed cohort, reductions in the integrity of left precuneus and superior parietal cortex originating white matter correlated with symptom severity. The authors noted that the microstructural integrity findings, in the context of covariance with comorbidities and associations with individual differences in severity, point towards neuroplastic phenomena – underscoring the need for longitudinal studies.

Lan et al. (2025) used magnetic resonance spectrography to study neurometabolite conditional dependencies within an anterior region of the default mode network, the SMA, and a posterior region of the default mode network in 32 children with FND and 41 HCs. Compared to HCs, the pediatric FND cohort showed that neurometabolites [creatine and glutathione] in the anterior default mode network displayed loss of conditional dependency with neurometabolities in the posterior default mode network and SMA. Given that glutathione and creatine are antioxidants, and that creatine plays a role in maintaining bioenergetics, the authors suggested that the findings point toward alterations in bioenergetics within the default mode network – suggesting potential difficulties with energy homeostasis and oxidative balance in pediatric FND.

## 4 Other articles

Lozzi et al. (2026) applied a seven-class microstate decomposition to electroencephalography data in 39 patients with FND-mixed and 47 HCs. Compared to HCs, the FND-mixed cohort exhibited a reduced duration of microstate G – which is associated with sensorimotor integration. A trend (inverse correlation) was observed between microstate G temporal stability and motor symptom severity. Additionally, several microstate transitions distinguished FND-mixed from HC cohorts. The authors suggested that the microstate G finding points towards altered maintenance of coherent somatosensory representations – underscoring that further characterization of microstates may provide a more comprehensive synthesis of the pathophysiology of FND.

Myren et al. (2026) advocated for the use of ultra-high field strength brain MRI research in FND. Such research would provide improved spatial resolution in the study of subcortical and brainstem structures (e.g., thalamus, locus coeruleus, periaqueductal gray, amygdala, hippocampus). The laminar architecture of the neocortex could also be better interrogated with high-field strength MRI, of relevance to FND research given conceptual models implicating aberrant predictive processing in this population.

## 5 Conclusion

In closing, this collection of articles addresses prior gaps in the FND literature. Observations continue to support that FND is in part a problem of alterations within and across multiple brain networks – and that these findings relate to individual differences. The data for FND representing a “software” *and* “hardware” problem of the brain is also growing – with many nuances emerging.

## Funding

None.

## Declaration of competing interest

D.L.P. has received honoraria for continuing medical education lectures in FND, royalties from Springer for a functional movement disorder textbook and honoraria from Elsevier for a functional neurological disorder textbook; is on the editorial boards of *The Journal of Neuropsychiatry and Clinical Neurosciences* (paid), *NeuroImage Clinical* (paid), *Brain and Behavior*, *Epilepsy & Behavior*, *Cognitive and Behavioral Neurology,* and *General Hospital Psychiatry*; has received funding from the Sidney R. Baer Jr. Foundation, Warren Alpert Foundation, and the National Institutes of Health unrelated to this work; and is on the FND Society Board and American Neuropsychiatric Association Advisory Council. All other authors report no conflicts of interest.
